# Potential Anaphylaxis to Systemic Phenylephrine: A Case Report

**DOI:** 10.1177/00185787231185867

**Published:** 2023-07-14

**Authors:** Sophia Pathan

**Affiliations:** 1 Hospital of the University of Pennsylvania, Philadelphia, PA, USA

**Keywords:** adverse drug reactions, allergy, medication safety

## Abstract

This case summarizes a 66 year old woman with a past medical history of asthma, breast cancer, anxiety, and chronic back pain with prior surgical insertion of spinal hardware, presenting for removal of spinal hardware at L5-S1 and spinal laminectomy and fusion at L4-L5. Her course was complicated by acute blood loss anemia and post-operative hypotension managed with a phenylephrine infusion, for which she experienced a possible anaphylactic reaction requiring upgrade to the intensive care unit. She was managed using intramuscular epinephrine, diphenhydramine, methylprednisolone, albuterol, famotidine, and oxygen. As phenylephrine is structurally similar to the endogenous catecholamines epinephrine and norepinephrine, diagnosis of her reaction was difficult. However, phenylephrine contains sodium metabisulfite, a preservative sulfite that may cause allergic-type reactions, particularly in patients with asthma in their medical history. Her hypersensitivity reaction was likely secondary to her use of phenylephrine, and she was discharged with a plan for outpatient follow up with the allergy team. As this case exemplifies, patients with a predisposition to allergic reactions should be closely monitored for possible hypersensitivity to preservatives and excipients in medications.

## Introduction

Sodium metabisulfite (SMS) is a preservative used in many cosmetic, food, and pharmaceutical products.^1,2^ It is one of the most frequently used sulfur-based molecules utilized as a preservative, and, as a sulfite, it can be affiliated with various allergies and allergic-type reactions.^1^ SMS has independently been affiliated with an asthma-like allergy that can cause asthma attacks with shortness of breath, wheezing, cough, and chest tightness. Additionally, patients have been reported to experience hypersensitivity reactions to SMS itself. The overall prevalence of sulfite sensitivity in the general population is unknown and probably low, but it is seen more frequently in asthmatic than in nonasthmatic people.^1,2^

A 2012 retrospective case series and literature review revealed an increasing prevalence of reactions to SMS in a patch-test.^3^ In this series, 4.5% of patients tested experienced a reaction, noting that the most frequent localizations of patch-test positive lesions were the face and hands, with some patients experiencing systemic symptoms.^3^ Thirty-three medication-related cases were found to cause reactions, including topical antifungals, topical steroids, topical anesthetics, local anesthetics, and eye drops.^3^ However, this series focused mainly on contact allergies to SMS, and reports of anaphylaxis are rare.

SMS is added as an excipient to medications which contain epinephrine and its derivatives in order to prevent the oxidation of the drug leading to its expiration.^1,2^ By preventing drug oxidation, the expiration date of the formulation is lengthened. Epinephrine derivatives include norepinephrine, phenylephrine, and dopamine. Phenylephrine, therefore, contains SMS, and use of phenylephrine has the potential to cause contact allergies and potentially anaphylaxis.

The most reported adverse reactions caused by phenylephrine are allergic contact dermatitis caused by eye drops,^4,5^ eardrops,^6^ or topical preparations.^7^ Although uncommon, phenylephrine sometimes has been found to cause a type IV hypersensitivity reaction, also known as cell-mediated or delayed-type hypersensitivity.^4^ Type I hypersensitivity reactions, also known as anaphylactic hypersensitivity reactions, are not documented as being triggered by phenylephrine. Still, a few case reports have found patients developing anaphylaxis to SMS, and as an ingredient in phenylephrine preparations, the potential for anaphylaxis to phenylephrine products cannot be ignored.^8,9^

This case documents a potential anaphylactic reaction to systemic phenylephrine likely secondary to the SMS preservative, and it highlights the importance of monitoring high risk patients, like asthmatics, who may experience hypersensitivity reactions to unlikely medications.

## Case Report

A 66 year old woman with a past medical history of hypertension, hypothyroidism, asthma, breast cancer (status post mastectomy, chemotherapy, and radiation in 2018), anxiety, depression, post-traumatic stress disorder, attention deficit disorder, and restless leg syndrome presented to the hospital for spinal surgery. Her home medications included alprazolam 1 mg daily, amphetamine-dextroamphetamine 10 mg twice daily, gabapentin 300 mg twice daily, levothyroxine 75 µg daily, montelukast 10 mg daily, pantoprazole 40 mg twice daily, rosuvastatin 10 mg daily, and valacyclovir 500 mg daily. Her allergy list included strawberries, cephalexin, and adhesive tape, for which she experienced itching and rash.

She presented with lumbar spondylosis and was brought to the operating room for removal of previous hardware and a lumbar fusion of L5-S1 and laminectomy of L4-5. While in the operating room, she received fentanyl 200 µg, lidocaine 100 mg, propofol 160 mg, ondansetron 4 mg, dexamethasone 4 mg, vancomycin 1 g, vecuronium 15 mg, ketorolac 15 mg, hydromorphone 2 mg, intravenous acetaminophen 1000 mg, and a phenylephrine infusion over 1 hour and 15 minutes for a total of 850 µg given.

Post-operatively, she was on the general neurology floor and reported incisional back pain and upper thigh pain but was overall hemodynamically stable. She was moving well, her sensations were symmetric bilaterally, and her incision site had mild bloody staining on the dressing but was healing appropriately. She had postural headaches that would resolve with pain medication administration such as with ketorolac and gabapentin.

Two days after her procedure, the patient was found to have confusion and hypotension around 07:49 am. Due to her hypotension, at 07:59 am, she was started on a phenylephrine infusion. Approximately 10 minutes after initiation of the phenylephrine infusion, the patient reported throat constriction, some shortness of breath, and some nausea. She also had a new rash on her neck and was experiencing itching in the area. Despite her symptoms, her oxygen saturation was noted to be 100%. On exam, her lungs were found to be clear to auscultation without tongue, tonsil, or uvula swelling.

At this time, it was determined that the patient’s symptoms, including cutaneous, gastrointestinal, and respiratory, were concerning for anaphylaxis. Approximate timing of all medications she received prior to her possible anaphylactic reaction are in Table 1. At 08:19 am, dexamethasone 10 mg was administered intravenously, and the patient initially clinically improved. However, at 09:10 she was found to have diffuse pruritus on her neck and a sensation of throat swelling and stridor. She also reported having nausea. Her phenylephrine drip was stopped due to concern for an allergic reaction, and she received epinephrine 0.3 mg intramuscularly twice, as well as 25 mg of diphenhydramine intravenously once. The otorhinolaryngology team was consulted and performed a nasopharyngolaryngoscopy (NPL), which noted moderate watery edema of aryepiglottic folds, edema of arytenoids/post-cricoid, and slight left vocal cord hemorrhage. From here, the patient was transferred to the neurosciences intensive care unit (ICU).

**Table 1.** Medication Timing.

Medication	Timing	Notes
Acetaminophen	05:45	received two doses of acetaminophen in preceding 24 hours; received a dose of this in the operating room; received on previous admissions
Gabapentin	05:45	received multiple doses of gabapentin in preceding 24 hours; home medication; received on previous admissions
Heparin	05:46	received a dose of heparin in preceding 24 hours; received in the preceding 48 hours in the operating room; received on previous admissions
Lansoprazole	06:05	received a dose of levothyroxine in preceding 24 hours; has used multiple medications of this class in the past (home medication)
Levothyroxine	05:47	received multiple doses of ketorolac in preceding 24 hours; home medication
Ketorolac	05:46	received doses of this in preceding 24 hours; continued to receive doses of this in the ICU and on the floor; received on previous admissions
Phenylephrine	07:59	received an infusion of phenylephrine in the preceding 48 hours in the operating room

On the neurosciences ICU, the patient was managed with supportive care. As the patient had hypotension before administration of the potential culprit medication (phenylephrine), assessing hypotension due to this medication was difficult. However, the patient’s symptoms showed improvement with intramuscular epinephrine, which supported an allergic reaction to the phenylephrine.

The Allergy and Immunology Team was also consulted and checking tryptase levels, as part of the diagnostic assessment to confirm anaphylaxis as the cause of the patient’s acute symptoms. The patient’s initial tryptase level was drawn about 2 hours after initiation of the phenylephrine infusion and it was within normal range at 8.0 µg/L (institution reference range <11 µg/L), though it would be expected to elevated in setting of an allergic reaction. Her repeated tryptase level of 4.2 µg/L, drawn 16 hours after the initiation of the phenylephrine infusion, was also within normal range.

The patient stabilized on the ICU on 2 L nasal cannula. She received diphenhydramine 25 mg orally every 4 hours for pruritus, famotidine 20 mg intravenously twice daily, methylprednisolone 60 mg intravenously every 6 hours, and continuous fluids. She also received albuterol inhalations as needed and lorazepam intravenously as needed for anxiety. The patient had no further need for epinephrine intramuscularly, and the patient’s blood pressure was managed with fluids rather than with vasopressors, as other peripheral vasopressors also had the SMS perseverative as an ingredient. The patient was re-scoped by otorhinolaryngology team, with findings of swelling superior to vocal cords and left vocal cord bruising, but the patient was otherwise stable. Speech, language, and pathology cleared her for a regular diet, and she was transferred back to the floor after being on the ICU for about 30 hours.

The next day, on post-operative day 5 and 1 day after her hypersensitivity episode, the patient was deemed stable for discharge. The rash and pruritus of her neck had subsided. The Allergy and Immunology team felt the patient was safe for discharge without a prescription for an epinephrine auto-injector, though the patient was recommended to follow up with the outpatient allergy team for further testing for SMS allergy.

## Discussion

Phenylephrine, due to its similarity in structure to the body’s naturally occurring catecholamines epinephrine and norepinephrine, is not considered a likely allergen in causing hypersensitivity reactions. Additionally, as epinephrine is utilized in anaphylaxis treatment, the likelihood of an anaphylactic reaction to phenylephrine, its structurally similar counterpart, is low. However, as there are documented hypersensitivity reactions to SMS in other formulations of phenylephrine, this patient’s reaction was a possibility.^3-8^

SMS is a preservative in epinephrine, norepinephrine, and phenylephrine infusions, and the vasopressor that is most structurally different from these agents that also does not have SMS is vasopressin.^10-17^ The infusion with the highest concentration of SMS is phenylephrine, making it more likely for patients to experience hypersensitivity reactions to this infusion compared to others.^10,11^

The epinephrine package insert explicitly states that the presence of bisulfite and sulfite as preservatives in the solution should not deter use in anaphylactic or allergic reactions.^12,13^ This was made clear during the patient’s case, as she was responsive to intramuscular epinephrine injections and experienced no additional allergy symptoms during epinephrine administration. Additionally, upon chart review of her prior surgical procedures and hospitalizations, she did not receive phenylephrine in the past but had received epinephrine as injections of lidocaine with epinephrine. If she needed a vasopressor for her blood pressure, either epinephrine or vasopressin would have been best to use. The epinephrine would help with anaphylaxis despite the SMS, and the vasopressin has no SMS so would not precipitate further anaphylaxis. The patient was responsive to fluids, however, and did not need further vasopressor for hemodynamic stability.

Still, this patient was predisposed to having an allergic reaction to SMS secondary to her past medical history of asthma.^1^ Per documentation in the electronic medical record, her first encounter with phenylephrine as a vasopressor was in the operating room for her surgical procedure on this admission, as her prior surgeries had not required phenylephrine. Her re-exposure to phenylephrine two days later represents a possible type I allergic reaction, with immunoglobulin E (IgE)-mediated hypersensitivity. Generally, this mechanism requires a first and then second exposure to the possible allergen. This patient experienced both a first and second exposure with the phenylephrine infusion in the operating room and then on the floor, as all other medications she received on the day of her reaction were ones she had either been receiving chronically or continued to receive throughout her hospitalization and to which she had no further reaction. Upon first exposure to the allergen, in this case the phenylephrine, the patient’s helper T cells stimulated the patient’s B cells to increase IgE production specific to the particular antigen, the SMS. On repeat exposure to the SMS via phenylephrine infusion, the SMS was able to bind to the IgE previously bound to mast cells, which could then be activated and cause the anaphylactic reaction with vasodilation secondary to histamines and prostaglandins. These inflammatory mediators are released by the mast cells, and activity of mast cells and mastocytosis can be measured using serum tryptase levels.

During treatment, tryptase measurement is a useful monitoring and prognostic tool.^18,19^ Tryptase is released into blood following mast cell activation. Total tryptase can be measured to confirm mast cell activation in diseases such as mastocytosis, anaphylaxis, urticaria, and asthma, but it is not generally used acutely except where diagnosis is unclear.^19,20^ Tryptase levels may correlate with mast cell burden, as mastocytosis can produce symptoms suggestive of organ involvement, such as enlargement of the liver, spleen, or lymph nodes. Measuring blood tryptase may be useful in the evaluation of patients with possible anaphylaxis or systemic mastocytosis.^19-21^ Following an anaphylactic episode, tryptase levels peak in 15 to 120 minutes and fall with a half-life of approximately 2 hours. In patients with systemic mastocytosis, tryptase concentrations are generally persistently elevated above 20 µg/L.^18,21^ Increased tryptase levels measured during a possible reaction to an allergen may suggest that mast cell activation may have had a role in causing the reaction.^19,20^

This patient’s initial and repeat tryptase levels were within the institution’s reference interval of <11.0 µg/L, which lowers the clinical suspicion for anaphylaxis due to lessened mast cell activation. However, it was possible in the setting of recent surgery and anemia that the patient’s threshold for allergic reaction was lowered. The lowered tryptase levels do make it difficult to confirm this patient’s diagnosis as hypersensitivity to SMS and phenylephrine, but clinical signs and findings for the diagnosis of anaphylaxis are still vital in diagnosis, as these are often the only available resources for diagnosis of anaphylaxis.

Rojas-Hijazo et al. described a patient who took a tablet with 10 mg of phenylephrine for a cold and experienced generalized urticaria, rhinoconjunctival edema, and eyelid edema. The patient experienced the reactions again during an oral challenge but not during a skin prick test, and they concluded their findings were related to an IgE-mediated mechanism.^9^ Additionally, Kendigelen et al reported anaphylaxis after administration of SMS-containing amikacin in a premature newborn, showcasing an IgE-mediated hypersensitivity reaction and likely a type I anaphylaxis reaction to SMS.^8^ These cases align with a likely anaphylactic reaction in this patient, rather than the type IV hypersensitivity reactions with contact dermatitis that prior case reports have detailed.^3-7^

As untreated anaphylaxis can be fatal, it is imperative that patients are appropriately assessed for allergies to SMS, even though hypersensitivity to phenylephrine, epinephrine, or norepinephrine is not frequently reported and has a low likelihood of occurrence. As such, on the Naranjo algorithm, or Adverse Drug Reaction Probability Scale, this patient scores a 5 for a probable adverse drug reaction.^22^ There are previous reports of reactions to phenylephrine, this event occurred after the medication was administered and improved with anaphylaxis treatment, and the patient presented with clear signs and symptoms of an allergic reaction. Unfortunately, this reaction was not re-tested with the same or similar medications, the dose was not adjusted to test the reaction, no placebos were given, and the drug was not re-administered to test the cause of the reaction.

To date, this is one of the only case reports to detail a possible type I hypersensitivity reaction to systemic phenylephrine secondary to its preservative SMS. This patient was high risk secondary to her asthma history, and her future use of vasopressors will need to be regularly assessed.

## Conclusion

Sodium metabisulfite is a preservative present in many intravenous medications, including phenylephrine infusions, and it can be the cause of anaphylaxis in high risk patients. Patients with a predisposition to allergic reactions should be closely monitored for possible hypersensitivity to preservatives and excipients in medications.
